# Hedgehog Autoprocessing: From Structural Mechanisms to Drug Discovery

**DOI:** 10.3389/fmolb.2022.900560

**Published:** 2022-05-20

**Authors:** Nabin Kandel, Chunyu Wang

**Affiliations:** ^1^ Department of Biological Sciences, Center for Biotechnology and Interdisciplinary Studies, Rensselaer Polytechnic Institute, Troy, NY, United States; ^2^ Department of Chemistry and Chemical Biology, Center for Biotechnology and Interdisciplinary Studies, Rensselaer Polytechnic Institute, Troy, NY, United States

**Keywords:** drug discovery, cholesterolysis, hedgehog autoprocessing, hedgehog signaling, inhibitor

## Abstract

Hedgehog (Hh) signaling plays pivotal roles in embryonic development. In adults, Hh signaling is mostly turned off but its abnormal activation is involved in many types of cancer. Hh signaling is initiated by the Hh ligand, generated from the Hh precursor by a specialized autocatalytic process called Hh autoprocessing. The Hh precursor consists of an N-terminal signaling domain (HhN) and a C-terminal autoprocessing domain (HhC). During Hh autoprocessing, the precursor is cleaved between N- and C-terminal domain followed by the covalent ligation of cholesterol to the last residue of HhN, which subsequently leads to the generation of Hh ligand for Hh signaling. Hh autoprocessing is at the origin of canonical Hh signaling and precedes all downstream signaling events. Mutations in the catalytic residues in HhC can lead to congenital defects such as holoprosencephaly (HPE). The aim of this review is to provide an in-depth summary of the progresses and challenges towards an atomic level understanding of the structural mechanisms of Hh autoprocessing. We also discuss drug discovery efforts to inhibit Hh autoprocessing as a new direction in cancer therapy.

## Introduction

### The Hedgehog Signaling Pathway Plays Essential Roles in Normal Physiology

Hedgehog (Hh) is a fundamental signaling pathway in metazoan development (Zeng et al., 2001; Ingham et al., 2011; Marini et al., 2011; Briscoe and Therond, 2013; Wu et al., 2017; Jiang, 2021). Hh was first identified in the fruit fly *Drosophila melanogaster* as an important morphogen in embryonic development in 1980s (Nusslein-Volhard and Wieschaus, 1980; Wieschaus, 1980). *Drosophila* Hh DNA was cloned in early 1990s (Lee et al., 1992; Tabata et al., 1992; Tashiro et al., 1993; Tabata and Kornberg, 1994) while the first human Hh gene was cloned in 1995 (Marigo et al., 1995). Mutations in human Hh gene leads to congenital diseases, such as holoprosencephaly (HPE), a congenital disease with impaired development of the midline structures of the brain (Belloni et al., 1996; Roessler et al., 1997; Nanni et al., 1999; Hehr et al., 2004). Three types of Hh genes have been discovered in vertebrates: *Sonic*, *Desert*, and *Indian*. Among the three, *Sonic* Hh is the best studied and acts as a morphogen in the patterning of almost every organ (Varjosalo and Taipale, 2008; Rimkus et al., 2016). *Sonic* Hh pathway also plays crucial roles in stem cell maintenance, tissue repair, and regeneration in adults (Ingham et al., 2011; Briscoe and Therond, 2013).

### The Hh Autoprocessing Generates Cholesterol-Modified Hh Ligand

Hh signaling is initiated by the Hh ligand, a product of specialized auto-catalytic process called Hh autoprocessing (Porter et al., 1996a; Hall et al., 1997; Xie et al., 2015; Smith et al., 2020). Hh ligand is generated from a precursor protein, composed of an N-terminal signaling domain (HhN) and a C-terminal autoprocessing (HhC) domain. HhN is preceded by a signaling sequence (SS) which targets Hh to endoplasmic reticulum (ER), where Hh autoprocessing occurs. The SS is cleaved off by a SS protease, leaving an N-terminal cysteine for later palmitoylation. HhC contains the Hint domain (Hedgehog/intein), an enzymatic module shared by Hh and inteins (Hall et al., 1997), and the sterol recognition region (SRR), which binds cholesterol. During Hh autoprocessing, the precursor is cleaved between N- and C-terminal domain followed by the covalent ligation of cholesterol to the C-terminal residue of HhN, which subsequently leads to the generation of Hh ligand, essential for the full activity of Hh morphogens (Lewis et al., 2001) and for Hh signaling (Porter et al., 1996b). The cholesteroylated HhN is then modified by palmitate at its N-terminal cysteine, catalyzed by Hh acyl transferase (HHAT) (Mann and Beachy, 2004; Jiang et al., 2021). The double lipidation of Hh ligand is crucial for both its transport and signaling potency (Chamoun et al., 2001; Gallet et al., 2003). Hh ligand is then secreted out of the cell and binds to the Patched (PTCH) receptor in target cells. Patched activation relieves its inhibition on Smoothened (SMO), another membrane protein, which turns on glioma-associated oncogene transcription factor (GLI) family of transcription factors to activate Hh signaling. Hh autoprocessing is important because it is at the origin of canonical Hh signaling, where it precedes all downstream signaling events (Porter et al., 1996a; Hall et al., 1997; Jiang and Paulus, 2010; Xie et al., 2014; Xie et al., 2015; Xie et al., 2016; Zhang et al., 2019; Zhao et al., 2019; Smith et al., 2020), and is unique to Hh proteins. Although it lies at the very origin of Hh signaling, there are only a few structural/mechanistic studies (Owen et al., 2015a; Owen et al., 2015b; Callahan and Wang, 2015; Bordeau et al., 2016; Ciulla et al., 2018; Ciulla et al., 2019; Zhang et al., 2019; Zhao et al., 2019; Smith et al., 2020), compared to the great number of studies of downstream components, such as PTCH (Ingham et al., 1991; Chen and Struhl, 1996; Sidransky, 1996; Kallassy et al., 1997; Xie et al., 1997; Zedan et al., 2001; Shao et al., 2006; Lorberbaum et al., 2016; Tukachinsky et al., 2016; Zhang et al., 2018; Abd Elrhman and Ebian, 2019; Kinnebrew et al., 2021), SMO (Xie et al., 2014; Owen et al., 2015a; Owen et al., 2015b; Callahan and Wang, 2015; Xie et al., 2015; Xie et al., 2016; Zhang et al., 2019; Zhao et al., 2019; Smith et al., 2020), and GLI (Lauth et al., 2007; Kim et al., 2010; Maun et al., 2010; Beauchamp et al., 2011; Pan et al., 2012; Long et al., 2016; Xiao et al., 2017; Kowatsch et al., 2019; Liu et al., 2019; Quaglio et al., 2020; Tran et al., 2020). In some context with direct cell-to-cell contact, the unprocessed full-length Hh protein is reported to have signaling activity (Tokhunts et al., 2010; Leprieur et al., 2017).

### Abnormal Activation of Hh Signaling is Linked to Cancers

Abnormal Hh activation plays an important role in many types of human cancers (Wellbrock et al., 2015; Cortes et al., 2019; Terao and Minami, 2019; Xie et al., 2020). Hh pathway also regulates cancer stem cells (CSCs) in breast cancer, pancreatic cancer, colon cancer, glioblastoma, multiple myeloma (MM), and chronic myeloid leukemia (CML) (Bar et al., 2007; Blotta et al., 2012). Hh pathway activation in cancer may be Hh ligand-independent or ligand-dependent. Ligand-independent pathway activation is characterized by loss-of-function mutations in the negative regulators such as PTCH or gain-of-function mutations in the positive regulator SMO, e.g., in basal cell carcinoma (BCC) (Reifenberger et al., 1998; Xie et al., 1998) and medulloblastoma (Reifenberger et al., 1998; Teglund and Toftgard, 2010). In Hh ligand-dependent pathway, the autocrine activation occurs when Hh ligand, produced by the tumor cell, activates Hh signaling in the same cell. Similarly, paracrine activation occurs when Hh ligand secreted by tumor cells turns on Hh signaling in the surrounding stroma. The tumor stroma then stimulates growth of the tumor. Inverse paracrine signaling occurs when stroma-derived Hh ligand activates Hh signaling in the tumor (Amakye et al., 2013). Many sporadic cancers are dependent on Hh ligand, and therefore may be sensitive to inhibitors of Hh autoprocessing. These include prostate cancer (Karhadkar et al., 2004; Sanchez et al., 2004; Sheng et al., 2004), lung cancer (Watkins et al., 2003; Velcheti and Govindan, 2007), pancreatic cancer (Thayer et al., 2003; Cengel, 2004; Kelleher, 2011; Bai et al., 2016), breast cancer (Tokhunts et al., 2010; Ali, 2012; Habib and O'Shaughnessy, 2016; Bhateja et al., 2019), and ovarian cancer (Chen et al., 2007; Liao et al., 2009). In prostate cancer, Hh ligand increases tumor growth and invasiveness, likely mediated by tumor-to-stroma communication (Fan et al., 2004; Yauch et al., 2008). In medulloblastoma, Hh ligand secreted by astrocytes also appears to drive tumor growth (Liu et al., 2017). Tumor-derived Hh ligand also plays an immunosuppressive role in breast and liver cancer, deterring infiltration of anti-tumor CD8^+^ T-cells (Petty et al., 2019). In multiple myeloma (MM), secreted Hh ligand through autocrine promotes tumor cell proliferation while enhancing chemoresistance (Liu et al., 2014). *For a subset of Hh ligand-dependent cancer, inhibitors of Hh autoprocessing have the potential of developing into a novel class of anti-cancer drugs.*

## Structural Mechanism of Hedgehog Autoprocessing

### Structural Mechanism of Hh Hint Domain

**FIGURE 1:**
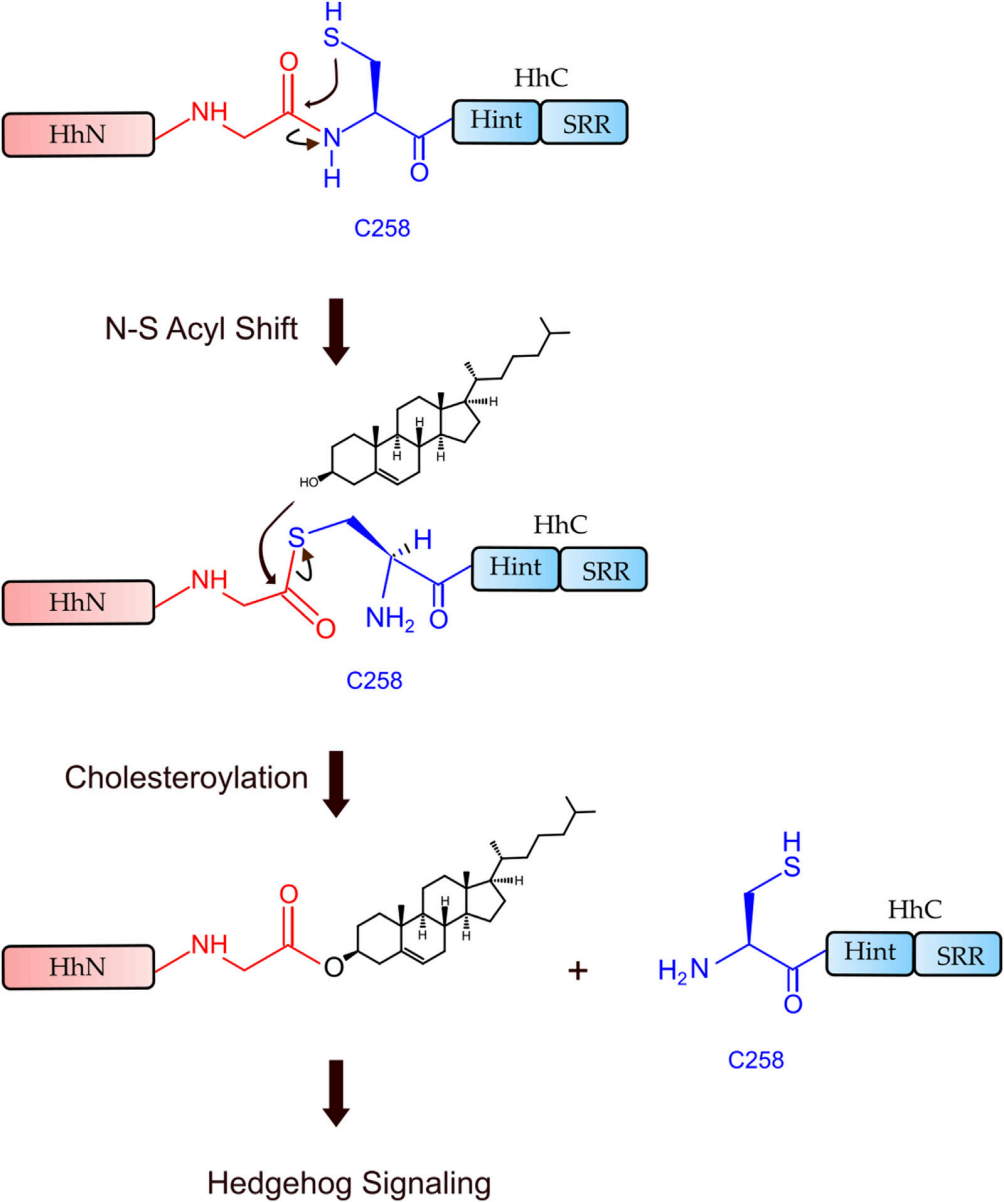
Hh autoprocessing. Hedgehog autoprocessing and the generation of Hh ligand involves two key steps: *N-S Acyl Shift* and *Transesterification*.

Hh Hint domain, and self-splicing inteins share several similarities in terms of their sequence, structure, and function, suggesting a common evolutionary ancestor (Koonin, 1995; Hall et al., 1997; Pietrokovski, 1998). Hh autoprocessing occurs in following two steps (Figure 1): 1) *N-S Acyl Shift*: C258 of HhC carries out a nucleophilic attack on the carbonyl of the last glycine (G-258) residue of HhN, resulting in a thioester intermediate. 2) *Transesterification*: The hydroxyl group of cholesterol non-covalently bound to the sterol recognition region (SRR) of HhC carries out a nucleophilic attack on the thioester, releasing HhN and linking it covalently to cholesterol (Ciulla et al., 2019). Inteins, on the other hand, catalyze protein splicing processes in four steps (Wall et al., 2021), with the first two steps very similar to Hh autoprocessing (Koonin, 1995; Hall et al., 1997; Pietrokovski, 1998), except that the intramolecular transesterification in intein splicing is replaced by an intermolecular transesterification, namely cholesteroylation of HhN in Hh proteins (Porter et al., 1996b). While Hh Hint and inteins have conserved C258 and TXXH motif in common, Hh Hint has unique residues not present in inteins, e.g., D303 and C400 *Drosophila* Hint domain.

**FIGURE 2:**
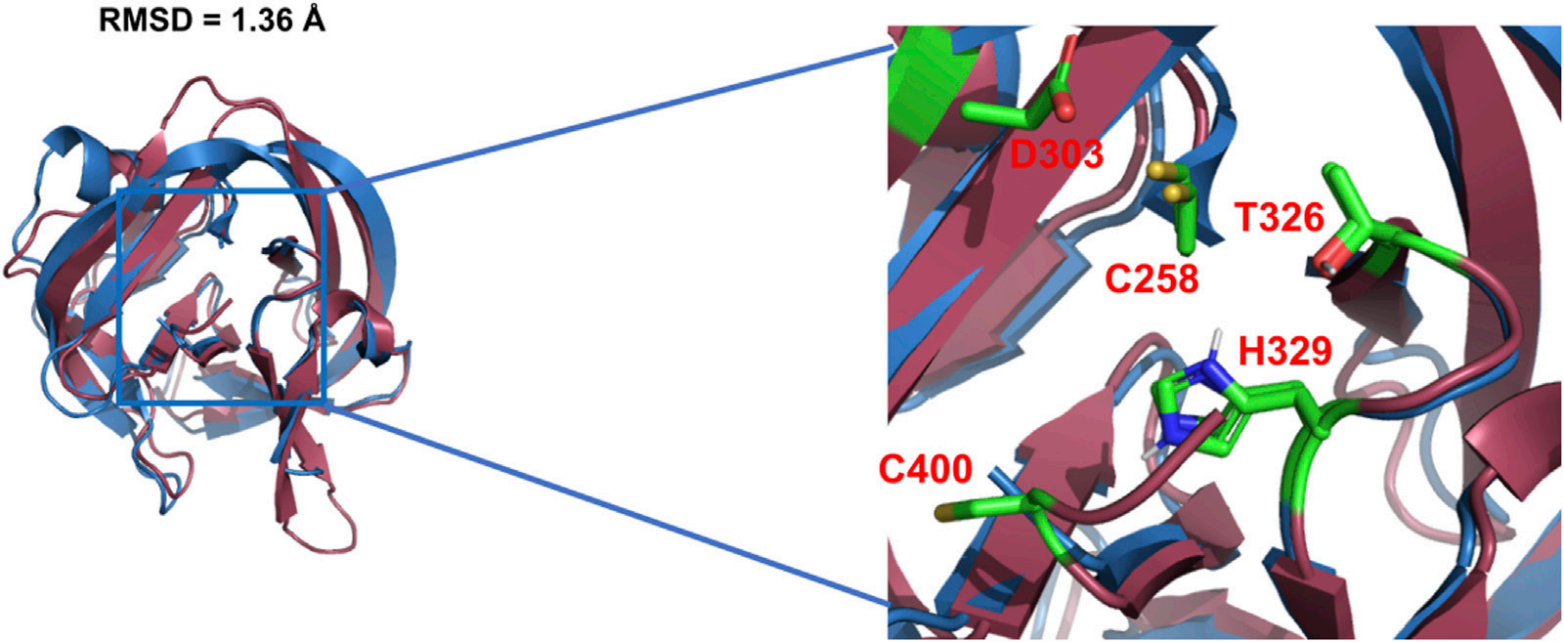
3D structures of Hh Hint and intein. Crystal structure of *Drosophila* Hh Hint domain (red, PDB: 1AT0) and intein (blue, PDB: 2IN0; an engineered mini-intein based on *Mtu* RecA intein) have similar 3D structures (RMSD = 1.36 Å). The image on the right zooms in the conserved residues in the active site of Hint and intein.

Using X-ray crystallography and mutagenesis, Hall et al. solved the first crystal structure of WT Hh Hint domain from *Drosophila* (Figure 2, **red**) and identified several crucial residues in Hh autoprocessing (Hall et al., 1997). As expected, Hh Hint shares a common fold as inteins (Figure 2, **blue**). The 3D structure is disc-shaped and is composed of all *β*-strands, with a diameter of ∼ 35 Å and width of ∼ 20 Å. Similar to inteins, the amino- and carboxyl-termini are close to each other, with only ∼ 6 Å apart. The side chains of conserved residues T326 and H329 in the conserved TXXH motif were found to be within H-bonding distance of C258. The involvement of active site residues D303, T326, and H329 in Hh autoprocessing was assessed by alanine mutations. The H329A mutant was found to be inactive in both DTT- and cholesterol-mediated reactions. Similarly, T326A mutant’s activity was also greatly reduced in both reactions confirming their catalytic roles. In contrast, D303A mutant showed active DTT-mediated N-terminal cleavage but was not able to mediate full cholesteroylation, underlying the unique role of D303.

**FIGURE 3:**
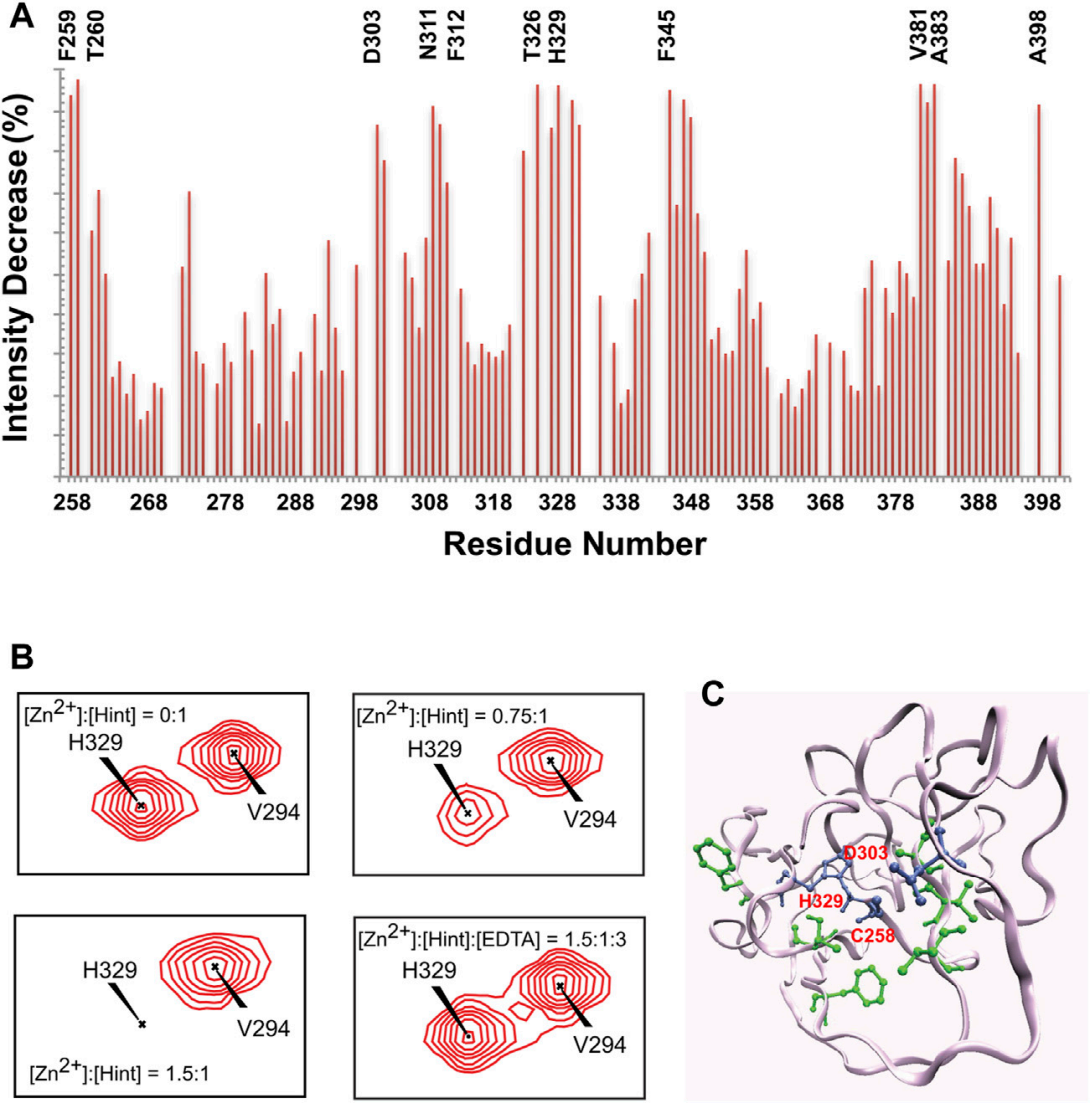
Structural basis of zinc-Hint binding. **(A)** ^1^H-^15^N HSQC signal intensity changes of Zn^2+^ binding to Hint ([Zn^2+^]:[Hint] = 1.5:1) at 25°C, residues with biggest changes labeled. **(B)** Chemical shift perturbation analysis for H329 backbone in ^15^N-labeled Hint caused by zinc binding. The amide peak intensities of H329 decreased with increasing zinc concentration. Missing signals were restored with addition of 2 molar equivalent of EDTA. **(C)** Structural model of Hint binding site of Zn^2+^ mapped onto the x-ray structure (PDB: 1AT0) based on the NMR signal intensity change. Blue residues indicate direct coordination sites, with the biggest signal decrease while green residues, with less signal reduction, likely play secondary role in zinc binding. Figure from the reference, Xie et al. (Xie et al., 2015) with permission.

***Zinc.*** Hh autoprocessing has been shown to be inhibited by zinc (Xie et al., 2015), similarly to intein-mediated protein splicing (Hall et al., 1997; Zhang et al., 2009) at protein level. Zinc deficiency and overproduction of Hh ligand has been demonstrated to co-exist in many types of cancers including prostate cancer, lung cancer, and ovarian cancer (Costello and Franklin, 2011; Zhang et al., 2012; Bjorklund, 2013). Xie et al. showed that zinc inhibits Hh autoprocessing with µM efficacy both *in vitro* and in cells (Xie et al., 2015). Using chemical shift perturbations (CSP) in ^1^H-^15^N HSQC spectra, they identified the residues C258, D303, and H329 as major coordination sites of Zn in the Hint domain (Figure 3). Thus, Zn binding likely inhibits Hh autoprocessing by perturbing their active site geometry and mobility ***.***

### Structural Mechanism of Conserved Residue 303 in *Drosophila* Hint Domain

#### Pivotal General Base D303 in HhC Autoprocessing

**FIGURE 4:**
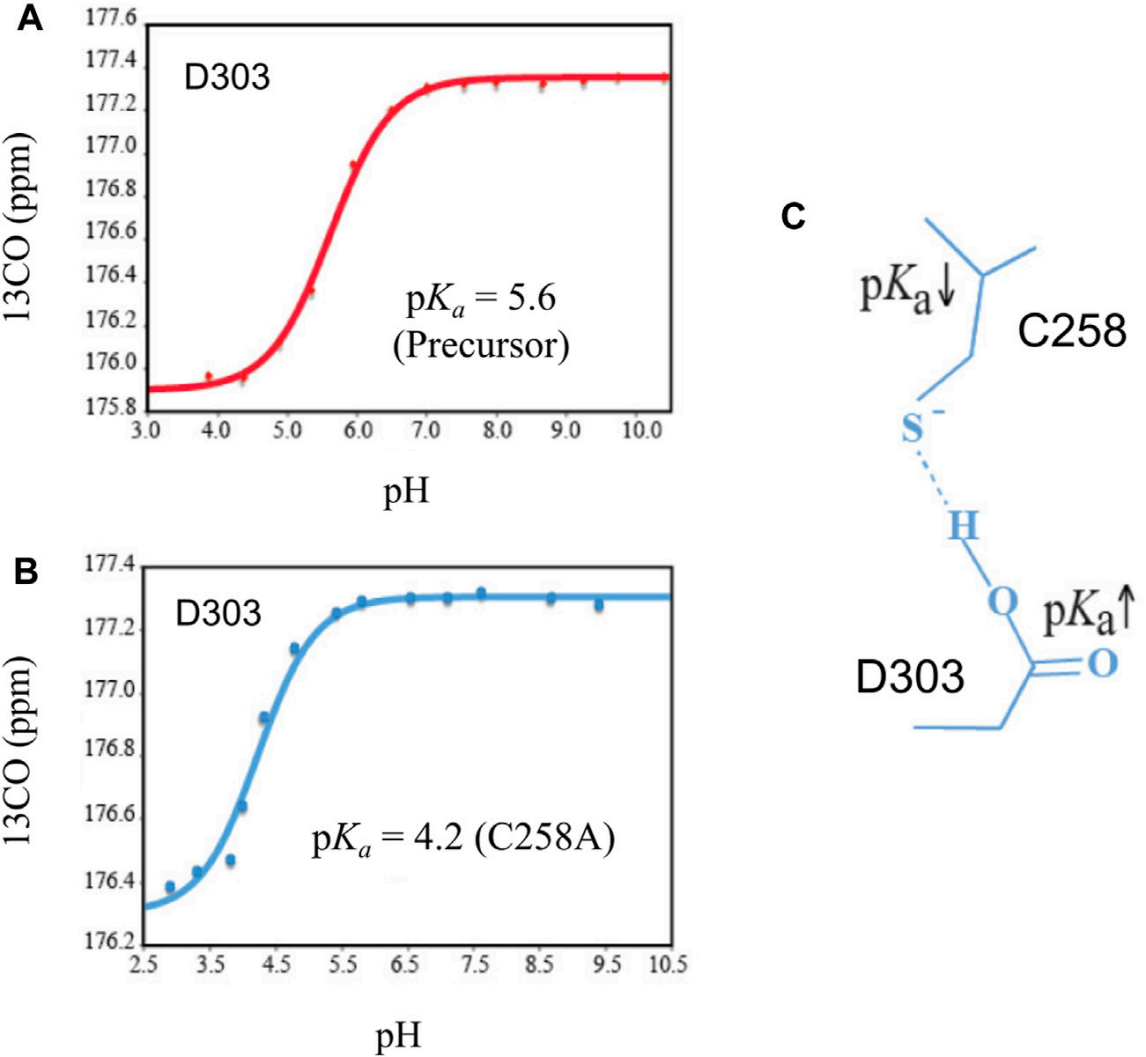
Coupling of pKa between D303 and C258. **(A)** The p*Ka* of D303 is increased to 5.6, as determined by ^13^CO chemical shift titration with HB(CB)CO. **(B)** C1A mutation brings the p*Ka* of D303 back to normal. **(C)** The structural basis of p*Ka* shift and coupling between C258 and D303. Figure from the reference, Xie et al. (2016) with permission.

**FIGURE 5:**
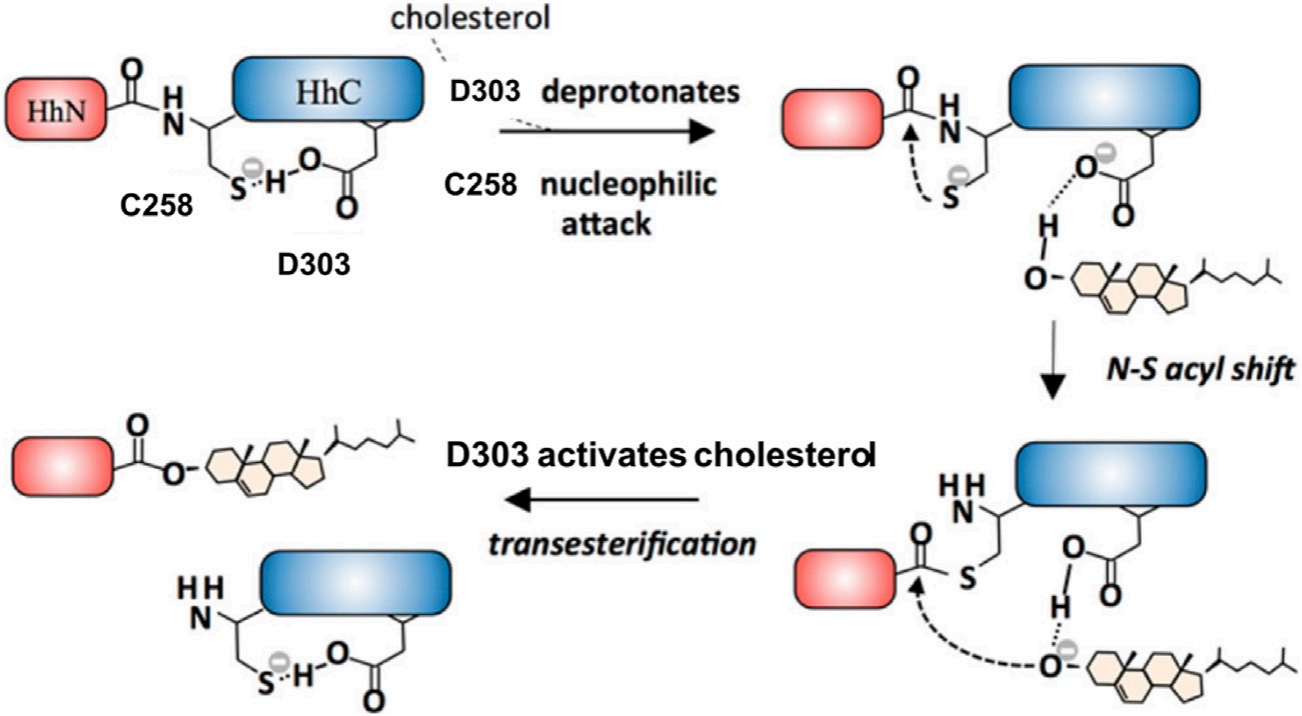
D303 coordinates two key steps of Hh autoprocessing. Coordination mechanism of two key steps of Hh autoprocessing by conserved D303 residue in Hint. Figure from the reference, Xie et al. (Xie et al., 2016) with permission.

The general base D303 (D46 in Hint numbering) has been shown to coordinate the two very important catalytic steps in Hh autoprocessing (Xie et al., 2016). Through NMR analyses, Xie et al. found that in the wild-type protein, the p*K*_*a*_ value of D303 side chain is significantly elevated by 2 pH units or 100-fold (Figure 4A) while in C258A mutant, D303 has normal p*Ka* of 4.2 (Figure 4B), indicating a p*Ka* coupling and a hydrogen bond between D303 and C258 (Figure 4C). A catalytic proton shuttling mechanism has been proposed in which D303 coordinates the two steps of Hh autoprocessing including the critical deprotonation of substrate cholesterol (Figure 5). In this mechanism, the D303 carboxyl first holds C258 thiolate in an inactive conformation, supported by the p*Ka* measurement and the finding that DTT-mediated cleavage becomes more efficient with D303 mutations D303A, D303N, D303R, and D303E. This prevents premature N-S acyl shift and non-productive precursor hydrolysis. When D303 deprotonates, C258 thiolate becomes free to carry out the nucleophilic attack on C258 carbonyl for N-S acyl shift. At the same time, deprotonated D303 sidechain interacts with the hydroxyl of cholesterol, deprotonating it and activating the cholesterol hydroxyl group to attack the thioester.

#### Mechanisms of D303H Variant

**FIGURE 6:**
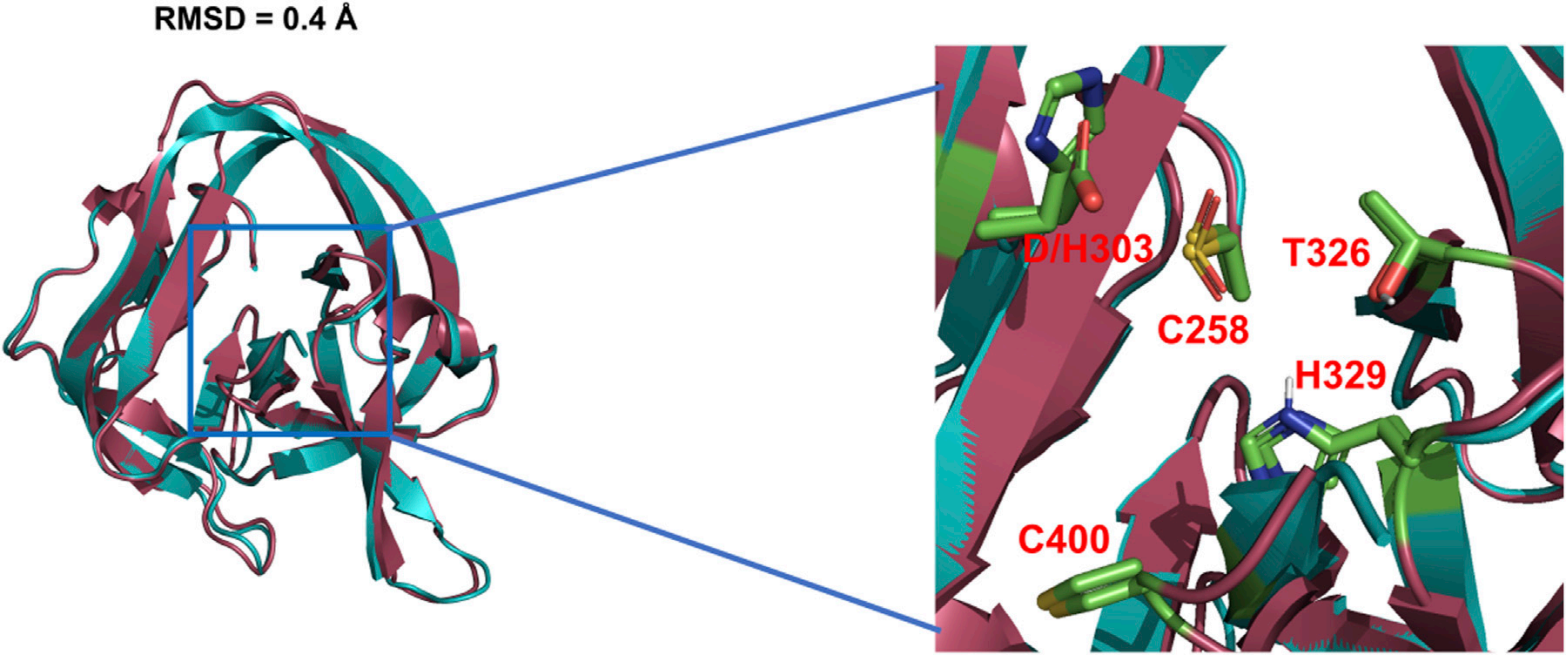
3D structures of WT Hh Hint and D303H. Crystal structures of WT Hh Hint domain (red, PDB: 1AT0) and D303H (cyan, PDB:6TYY) are almost identical (RMSD = 0.4 Å). The right image shows a magnified region of the conserved residues in the active site of WT and D303H.

**FIGURE 7:**
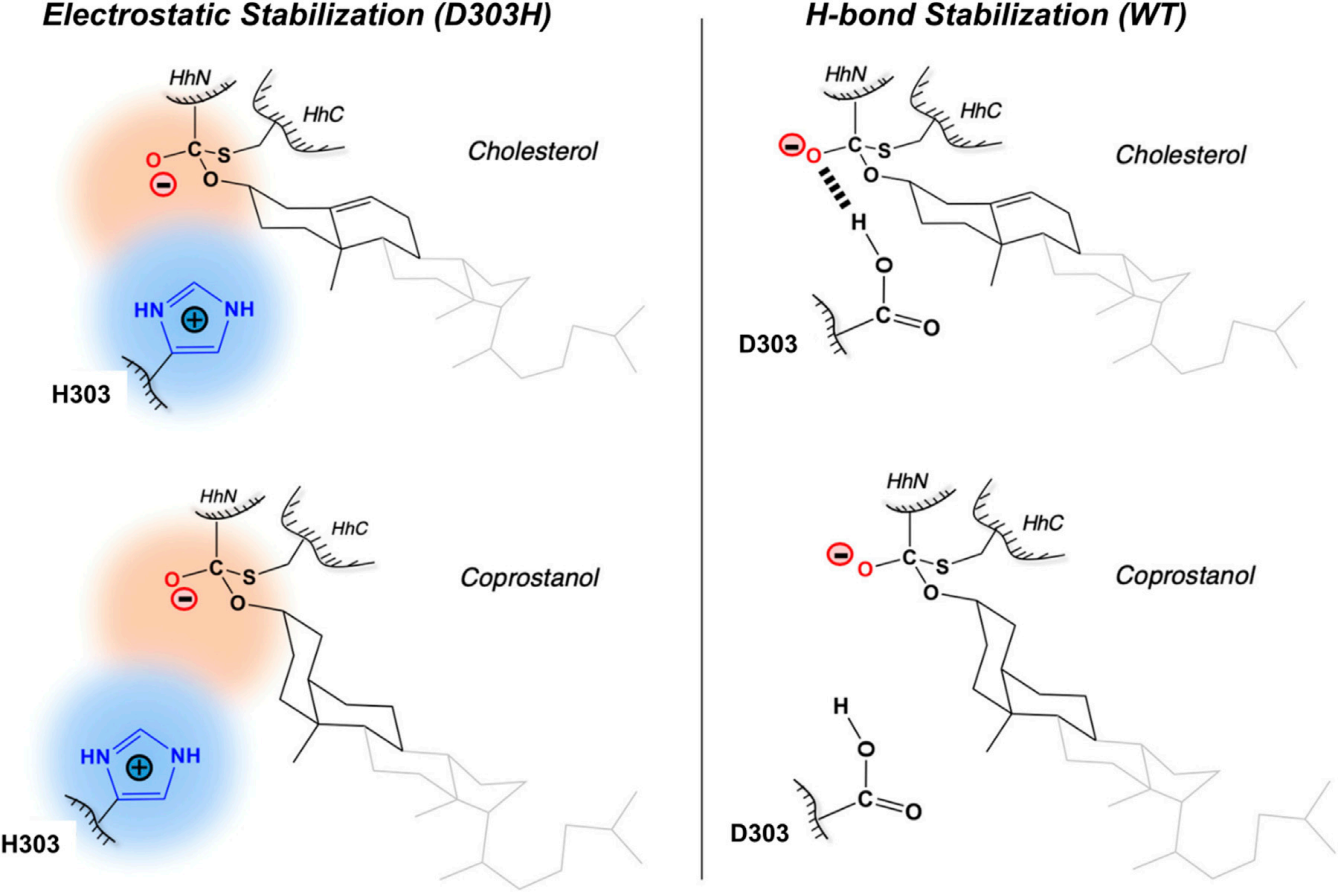
Electrostatic stabilization mechanism by H303. Mechanism of electrostatic stabilization by H303 sidechain not available in D303. H303 promotes formation of the negatively charged tetrahedral addition intermediate through favorable electrostatic interaction with its side chain imidazolium. Figure from the reference, Zhao et al. (2019) with permission.

The general base-swap D303H has been shown to preserve both structure and activity while also expanding substrate space in Hh autoprocessing (Zhao et al., 2019). Using X-ray crystallography, D303H Hint domain (Figure 6, **cyan**) was found to have an almost identical 3D structure compared to WT (Figure 6, **red**) with an RMSD of 0.4 Å. NMR pH titration showed that H303 has the same p*Ka* as D303. The similar p*K*_*a*_ values, 3D structures, and catalytic efficiency of the WT and D303H suggest that the D303H mutant shares a similar catalytic mechanism as the WT D303. Intriguingly, D303H exhibited enhanced catalytic activity toward non-native substrates, especially coprostanol (>200-fold) and epicoprostanol (>300-fold) (Zhao et al., 2019). Coprostanol, with a bent A-ring shows strong autoprocessing activity by D303H with a *K*_*M*_ of 2.4 µM and *k*_*max*_ of 2.9 × 10^−3^ s^−1^ compared to WT with a *K*_*M*_ of 25 µM and *k*_*max*_ of 0.3 × 10^−3^ s^−1^. Similarly for epicoprostanol, D303H has a reported *K*_*M*_ of 16 µM and *k*_*max*_ of 1 × 10^−3^ s^−1^ compared to no detectable reactivity of WT. This expanded substrate tolerance is likely due to stabilization by electrostatic interactions from H303 sidechain with less geometrical constrains than H-bonding stabilization by D303. The general base H303 promotes the formation of negatively charged tetrahedral addition intermediate through favorable electrostatic interaction with its side chain imidazolium (Figure 7) in the transition state. A notable finding from this study is that the sterol recognition is influenced by mutations in the Hint sub-domain. This indicates that the sterol recognition is shared between the SRR and the Hint sub-domains.

#### Chemical Rescue of D303A Mutants With Hyper-Nucleophilic Sterols

**FIGURE 8:**
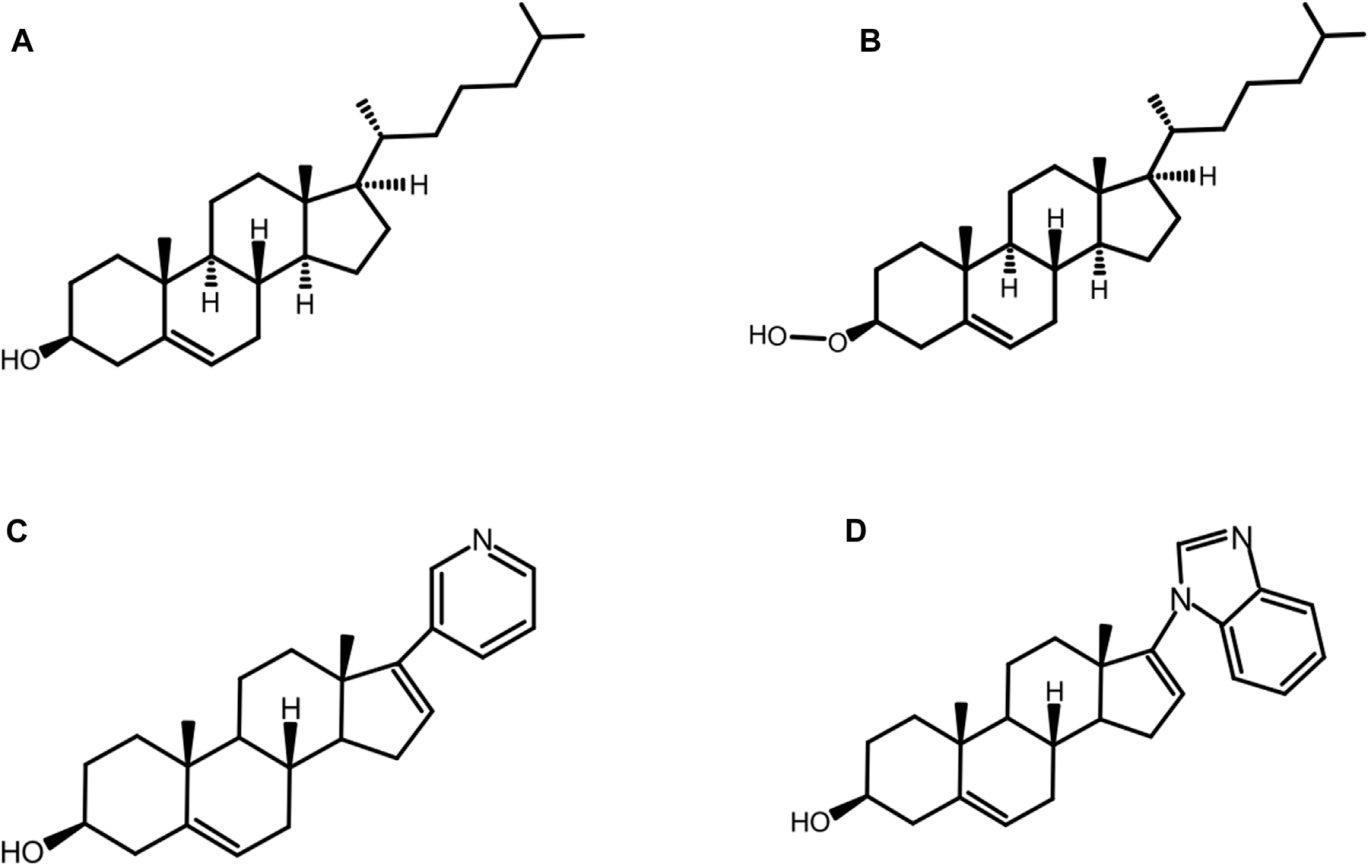
Native and non-native substrates. Structure of native substrate: cholesterol **(A)** and non-native substrates: 3-hydroperoxy cholestane: 3-HPC, a type of hyper nucleophilic sterol substrate **(B)**, abiraterone **(C)**, and galeterone **(D)** for HhC autoprocessing.

While the D303A mutation abolishes native autoprocessing activity, it has been observed that the mutant is *not* entirely catalytically inert. Ciulla et al. (Ciulla et al., 2018) chemically rescued autoprocessing in a cell free system with *D. melanogaster* HhC point mutant D303A by the synthetic substrate, 3*β*-hydroperoxycholestane (3HPC) (Figure 8B) where the −OH group of cholesterol (Figure 8A) is replaced by the hyper-nucleophilic −OOH group. The mutant D303A lacks the key general base necessary to activate the 3-OH of cholesterol. Efficient rescue of D303A by 3-HPC was described in terms of the “*α*-effect”, where tandem electronegative atoms like the peroxy group (HO-O-R) of 3-HPC exhibit exceptionally high nucleophilicity even at low basicity.

### Structural Mechanism of Cholesterol in Hh Autoprocessing

**FIGURE 9:**
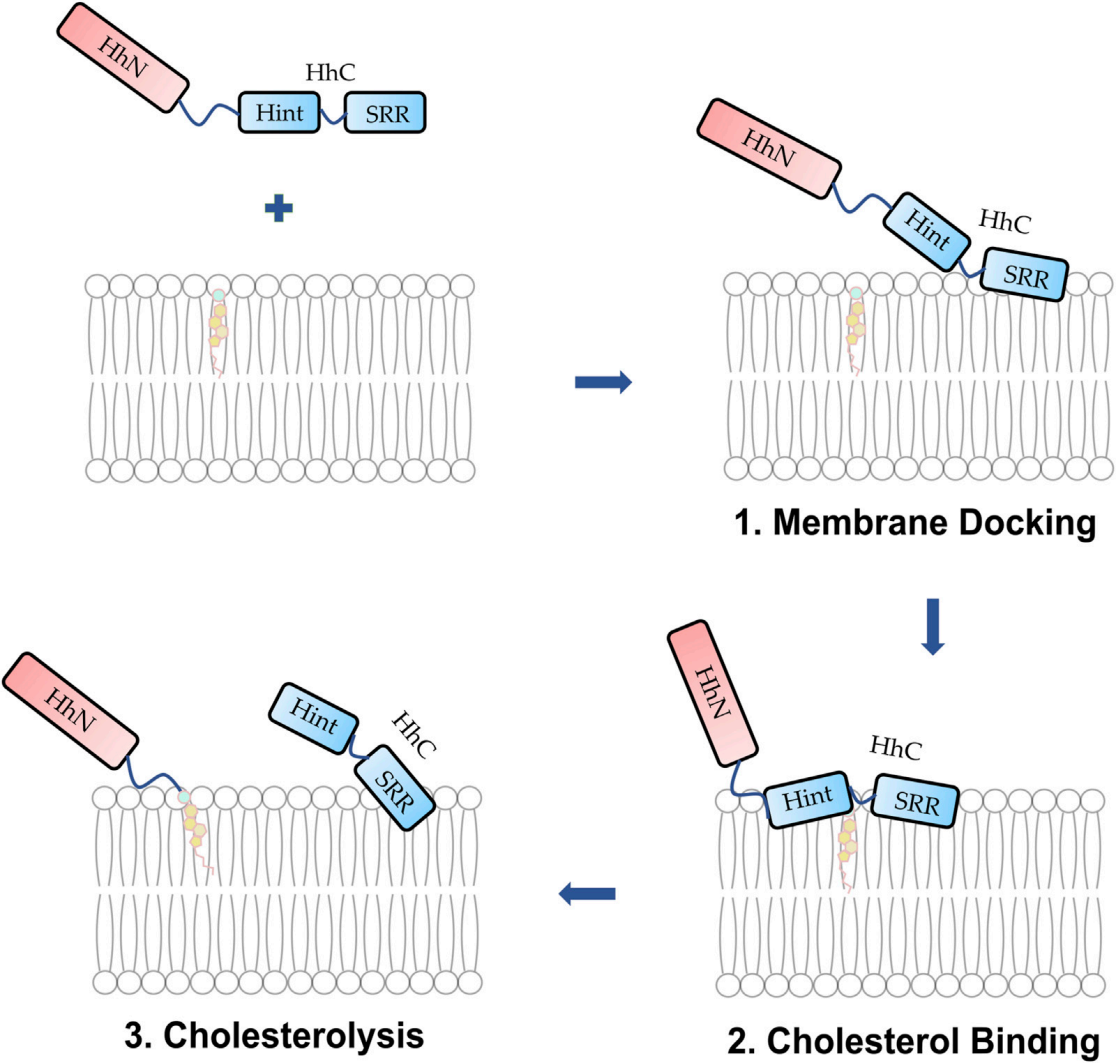
Proposed mechanism of membrane docking, cholesterol binding, and cholesterolysis. (**1**) The Hh protein docks to the membrane. (**2**) The SRR cooperates with the Hint enabling cholesterol to access the thioester. (**3**) Cholesterolysis proceeds followed by the generation of Hh ligand.

Cholesterol plays a key role in the biogenesis of Hh ligand during Hh autoprocessing (Figure 1). The marked insolubility and membrane sequestration of cholesterol requires its mobilization from the membrane (Figure 9) (Saad and Higuchi, 1965). Being a hydrophobic nucleophile with a p*Ka* of ∼ 18, the hydroxyl of cholesterol demands strict requirements on residues involved in cholesterol binding and nucleophilic attack. Detailed molecular mechanism on how Hh molecules access cholesterol in the membrane would be highly informative. Autocatalytic nature of HhC, presence of highly dynamic SRR, and challenges in protein overexpression and purification are some of the key factors impacting the experimental high-resolution structures of HhC and HhC-sterol complex. This has greatly hampered the understanding of how cholesterol interacts with Hh to enable Hh autoprocessing. However, MD simulations of HhC and HhC-sterol complexes have been carried out to understand HhC/cholesterol interaction, providing important insights and a framework for future experimental studies (Banavali, 2020; Mafi et al., 2021).

#### Cholesterol and HhC Residues Interactions

Using homology modeling based on *cryptogein* as a template for SRR, Banavali, N. K. derived the first structural model for SRR and cholesterol-bound Hh (Banavali, 2020). Restrained geometries and topology switches (RGATS) method was then used to derive a reaction pathway with atomic resolution. During the catalytic steps, no significant backbone structural changes in the Hh were observed, while both cholesterol and side chains of two catalytic residues G-258 and C258 changed their conformations. Almost all RGATS showed H-bonding between C258 side chain sulfur with the cholesterol O3 atom. Several other polar non-catalytic residues like T326 and H329 in TXXH motif, C400 in SCYA motif, Q446, and H450 in HWY motif were also observed to interact with the cholesterol. However, the simulation work was not carried out with lipid bilayer, the native environment for Hh autoprocessing.

#### Dual Role of SRR

Purohit et al. (2020) carried out the first extensive experimental characterization of SRR using human *Sonic* Hh (hSHH). A helix-loop-helix motif was proposed for SRR based on sequence analysis, modeling, and circular dichroism (CD) measurements. Mutagenesis was carried out to identify conserved residues which are important for Hh autoprocessing using both cellular and biochemical assays. Curiously, several residues, including L442, L446, and L450 located on the hydrophobic face of the 1st helix, are required for cholesterolysis in cells but *not* in *in vitro* biochemical assay. Deletion of the SRR 1st helix resulted in diffuse cytoplasmic distribution and loss of co-localization with Golgi organelle marker, indicating that these residues likely constitute a Golgi localization motif in cells. Thus, SRR not only interacts with cholesterol but also acts to direct Hh precursor to the correct cellular compartment.

#### Cholesterol Binds to Dynamic Hint-SRR Interface

**FIGURE 10:**
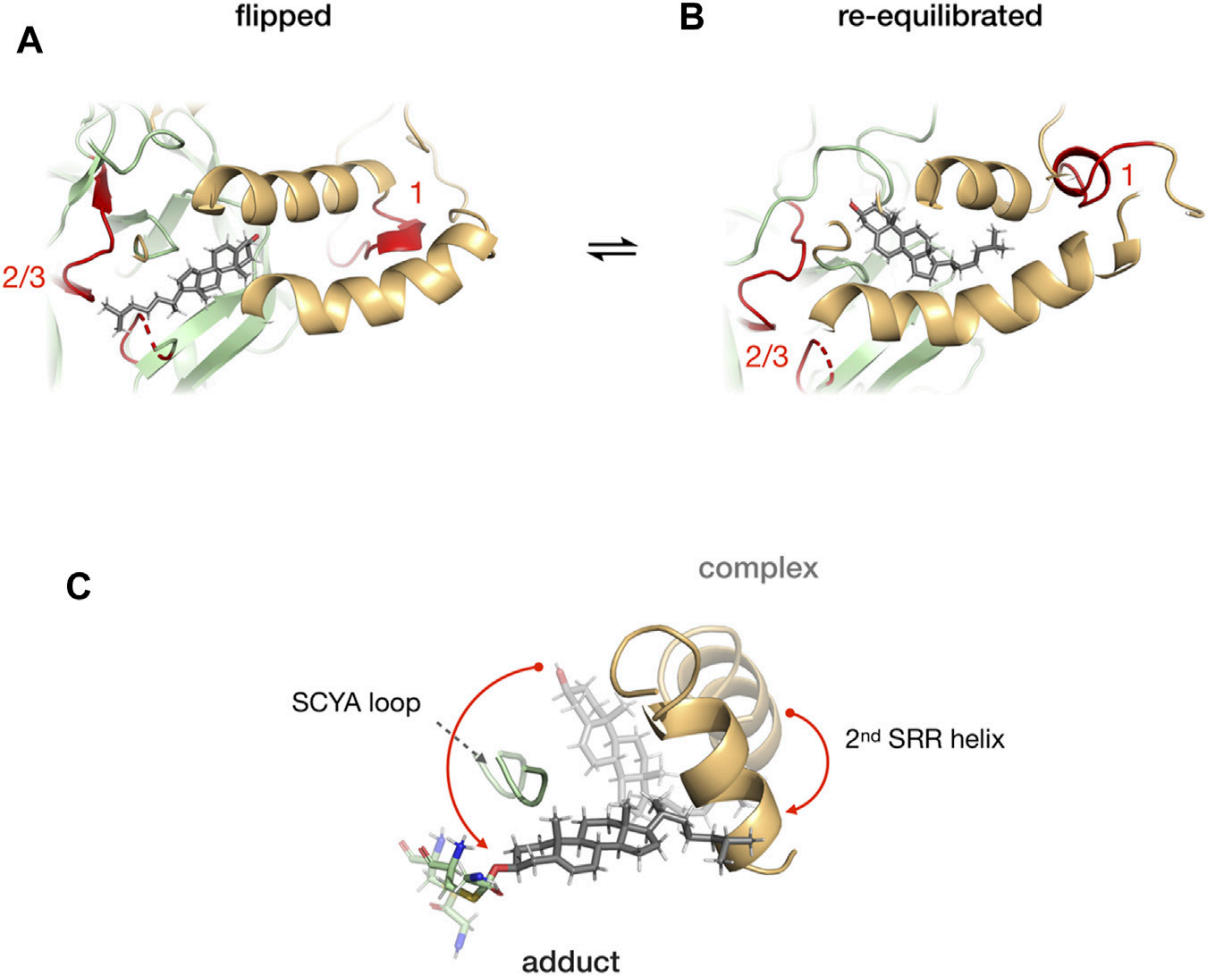
Cholesterol binding at the Hint-SRR interface. Dynamics of cholesterol-hog complex in flipped conformation where cholesterol molecule is rotated by 180° relative to its original position with binding site 1 from SRR and 2/3 from Hint (in red) **(A)**. The cholesterol returns to its original position in 400 ns MD simulation **(B)**. Overlay of cholesterol, 2nd helix, G258-C258 thioester, and SCYA loop from cholesterol-Hog complex and the Hog-cholesterol adduct **(C)**. The red arrow indicates the overall movement of the cholesterol. Figure from the reference, Mafi et al. (Mafi et al., 2021).

Mafi et al. (2021) combined molecular dynamics simulations, photoaffinity crosslinking, and mutagenesis to study SRR-cholesterol interactions in the hSHH protein. The MD simulations showed that the 1st helix can interact with membrane-bound cholesterol for recruitment while the 2nd helix can facilitate the re-orientation of cholesterol for the nucleophilic attack of the scissile bond carbonyl. The photo-cholesterol crosslinking identified three binding sites: site 1 in SRR, site 2 (C258) and 3 (T326 and H329 in TXXH motif) in Hint fold (Figure 10). A flipped conformation (Figure 10A) with cholesterol rotated by 180° was seen relaxing back to equilibrium position in 400 ns MD simulations (Figure 10B), indicating that the SRR-Hint interface can accommodate multiple binding modes of cholesterol. Thus, a hydrophobic Hint-SRR interface has been proposed that forms a dynamic, non-covalent cholesterol-Hog complex where Hh protein can access cholesterol. The migration of cholesterol to the active site residues caused large conformational changes in SRR (Figure 10C).

#### Abiraterone (Zytiga^TM^), a Robust Non-native Substrate for HhC Autoprocessing

The substrate tolerance of HhC is relatively broad, especially for native and non-native sterols. Mann and Beachy were the first to examine the structure activity relation (SAR) for cholesterol as the substrate for Hh autoprocessing (Mann and Beachy, 2004). The most important requirement is the presence and *β*-orientation of the 3-OH moiety, while other factors are not as crucial, such as the presence or absence of the isooctyl side chain, additional hydroxylation or conjugation. The steroidal anti-androgens, abiraterone and pre-clinical analog, galeterone, are some of the non-native counterparts that can replace cholesterol (Figure 8A) as substrate for HhC autoprocessing (Bordeau et al., 2016). Abiraterone (Figure 8C) and galeterone (Figure 8D) have been shown to activate Hh cholesterolysis. This non-native reaction generates *in situ* hedgehog drug conjugates, i.e., Hh-abiraterone. Bordeau et al. found that the off-target Hh-abiraterone generated by HhC stimulates the Hh pathway at low nanomolar concentration, on par with Hh-cholesterol (Bordeau et al., 2016). Because Hh signaling is deregulated in prostate cancer, and abiraterone is administered to treat advanced disease, this off-target HhC autoprocessing observed may have therapeutic significance.

#### Cholesterol A-Ring Analogues and Substrate Selectivity

In addition to cholesterol, a native substrate for HhC, there are several other non-native substrates, some of them with varying sterol A-ring plasticity (Ciulla et al., 2019). Ciulla et al. evaluated substrate activity of cholesterol (**I**), A-nor cholestanol, A-ring contracted sterol (**II**), A-ring fused sterol, a pentacyclic cholesterol derivative (**III**), and A-ring distorted sterol, non-planar cholesterol analogue, coprostanol (**V**). Interestingly, they reported the substrate selectivity among several geometric variants of sterol A-rings with relative reactivity in the order: cholesterol 1.000 > A-ring contracted 0.100 > A-ring fused 0.020 > A-ring distorted 0.005 (Ciulla et al., 2019). Although the detail mechanism of ∼ 100-fold reactivity difference among A-ring variants is not immediately clear, it strongly suggests that the active site of HhC puts a spatial restriction on cholesterol analogues and their activity.

## Drug Discovery to Inhibit Hedgehog Autoprocessing

Mechanistic understanding of Hh signaling has provided several antagonists that can block Hh signal transduction at various steps such as Hh acyltransferase inhibitors (Petrova et al., 2013), small molecule Hh inhibitors (Peng et al., 2009; Stanton et al., 2009), SMO inhibitors (Taipale et al., 2000; Chen et al., 2002; Williams et al., 2003; Song et al., 2019), and GLI inhibitors (Lauth et al., 2007; Hyman et al., 2009; Infante et al., 2015). Although several SMO inhibitors such as *vismodegib*, *sonidegib*, *and glasdegib* have been approved (Carpenter and Ray, 2019; Hoy, 2019), frequent SMO mutations have led to increased drug resistance demanding novel types of Hh inhibitors and rational multi-target combinations to overcome it (Metcalfe and De Sauvage, 2011; Katoh, 2019; Liu et al., 2019). Thus, compounds targeting Hh autoprocessing, the starting point of the Hh signaling pathway, will be of substantial merit to the drug candidate for combating abnormal Hh activation in many cancers.

Hh autoprocessing can offer an alternative target for the development of novel types of Hh inhibitors for therapeutic intervention in human cancers, particularly for Hh ligand-driven malignancies and where existing Hh blocking drugs have lost efficacy (Yauch et al., 2008; Amakye et al., 2013). Several HhC mutations found in congenital disease block autoprocessing and also suppress downstream signaling, providing an exciting potential for small molecule intervention for cancer therapy. Inhibiting the autoprocessing activity of HhC will block the biosynthesis of tumor derived bioactive Hh ligand, potentially exhausting a key oncogenic signal. Within the context of research works that have focused on the very origin of the Hh autoprocessing reaction, significant progress has been made in delineating the mechanism of Hh autoprocessing (Ciulla et al., 2018; Ciulla et al., 2019; Zhao et al., 2019; Banavali, 2020; Purohit et al., 2020; Mafi et al., 2021), and the development of various types of inhibitor and modulator compounds of this important catalytic process (Owen et al., 2015a; Owen et al., 2015b; Ciulla et al., 2019; Smith et al., 2020). These efforts have provided Hh autoprocessing as an alternative therapeutic target in cancer which can lead to the development of a novel class of anti-cancer agents.

### Covalent Inhibitor Compounds

#### Phenylarsine Oxide (PhAs^III^)

**FIGURE 11:**
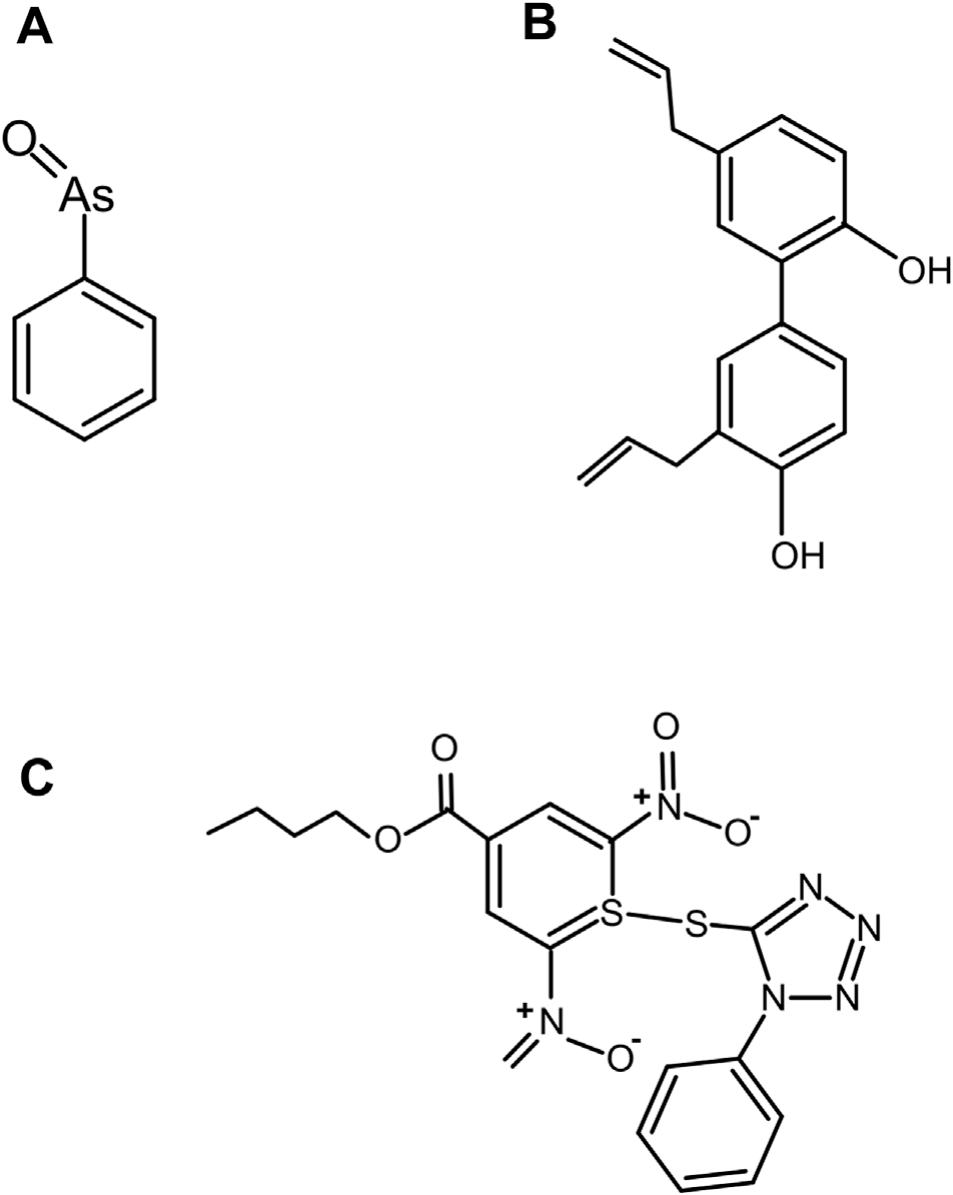
Covalent inhibitor compounds. Structure of covalent inhibitor compounds: *PhAs* ^*III*^ **(A)**, an antagonist of Hh cholesterolysis; *CID 72303* **(B)**, that can attenuate cholesterol-dependent Hh autoprocessing at micromolar concentrations as identified using HTS; and *ST044643* **(C)**, Hh cholesterolysis inhibitor compound*,* reported to inactivate Hh covalently using (S_N_Ar) mechanism as identified using FRET-based assay.

Previous research suggested the binding of As^III^ to Hh responsive transcription factor, GLI (Kim et al., 2010; Ng and Curran, 2011; Kim et al., 2013). Owen et al. (Owen et al., 2015b) used FRET based assay (Owen et al., 2015a) and demonstrated that trivalent arsenical compound, PhAs^III^ (Figure 11A) acts as a direct antagonist of Hh autoprocessing which can bind to Hh and irreversibly block the cholesterolysis (Owen et al., 2015b), with an IC_50_ of 2.2 µM and an apparent interaction constant of 0.4 µM. NMR titration demonstrated that PhAs^III^ binds to catalytic cysteines in the Hint domain. Thus, As^III^ compound may inhibit Hh signaling through interactions with multiple components within this signaling pathway.

#### Inhibitor Compounds CID 72303 and CID 5717

A high-throughput screen (HTS) assay was employed to identify compounds than can attenuate Hh autoprocessing (Jiang and Paulus, 2010). The HTS assay measures the changes in fluorescence polarization (FP) during the autoprocessing reaction. Jiang et al. used such an assay to identify the first two compounds, PubChem ID; *CID 72303* (Figure 11B) and *CID 5717* that can attenuate cholesterol-dependent Hh autoprocessing at micromolar concentrations (Jiang and Paulus, 2010). Both compounds inhibited cholesterol-dependent autocleavage, and not the hydroxylamine-dependent N-terminal cleavage suggesting that the inhibitors targeted the nucleophilic attack by cholesterol, instead of thioester formation. Time dependence of the inhibition suggests that *CID 72303* is a covalent inhibitor, while *CID 5717* is non-covalent.

#### Inhibitor Compound ST044643

*In vitro* focused library screening for Hh autoprocessing inhibitors have been employed to search for novel inhibitors of Hh autoprocessing among three commercial libraries of steroid-like compounds using FRET assay (Owen et al., 2015a). The central reagent for screening is an engineered Hh precursor, called C-H-Y, prepared by fusing cyan (C) and yellow (Y) fluorescent proteins to the N- and C-termini of *Drosophila* HhC. Strong FRET signal from precursor C-H-Y undergoes time-dependent loss in the presence of cholesterol. In the presence of an inhibitor, the FRET signal of C-H-Y decays more slowly or stays elevated. Using this assay, Owen et al. identified another novel Hh cholesterolysis inhibitor compound, *ID ST044643* (Figure 11C) with an IC_50_ of 5 µM (Owen et al., 2015a). This compound has been reported to inactivate Hh covalently by a substitution nucleophilic aromatic (S_N_Ar) mechanism.

### Non-Covalent Paracatalytic Activator Compound HAC8

**FIGURE 12:**
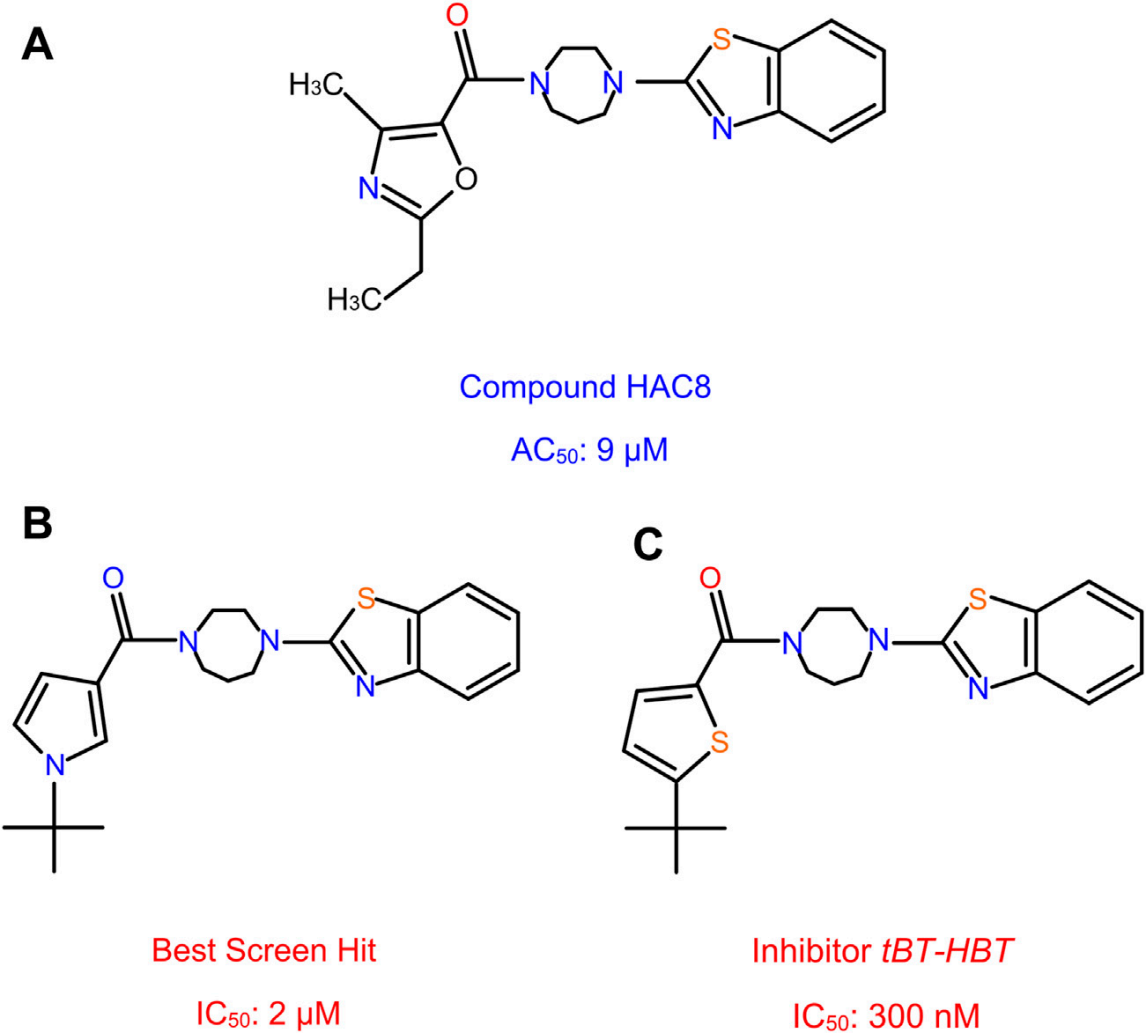
Non-covalent activator and inhibitor compounds. HAC8, a novel class of non-covalent modulator compound with an AC_50_ of 9 µM **(A)**. The best screen hit inhibitor compound with an IC_50_ of 2 µM **(B)**. The thiophene analog compound of the best screen hit, named *tBT-HBT*, the first nano-molar non-covalent inhibitor of HhC cholesterolysis, with an IC_50_ of 300 nM **(C)**.

Smith et al. (2020) discovered novel non-covalent modulators called HhC activator compound (HAC) which promotes the hydrolysis of Hh precursor, a side reaction of Hh autoprocessing, thereby inhibiting the cholesteroylation of Hh ligand. Using FRET-based assay, a focused library of 1187 steroid analogs were screened and identified molecules that influence HhC *via* an unusual and unexpected mode of action, termed paracatalytic induction (Figure 12A). When bound to HhC, these molecules activate autoproteolysis, a side hydrolysis reaction of HhC where the Hh precursor cleaves into HhN and HhC domains without the cholesteroylation of HhN. The most effective paracatalytic activator compound HAC8 has an AC_50_ of 9 µM and a corresponding *k*_max_ of 9 × 10^−4^ s^−1^.

### Non-Covalent Inhibitor Compound, tBT-HBT

More recently, Wagner et al. (Wagner et al., 2021) screened a library of sterol analogs and obtained the first nano-molar non-covalent inhibitor of Hh autoprocessing through structure activity relationship (SAR) of hit compounds. During the screening process, several hit compounds were identified, with the best screen hit having an IC_50_ of 2 µM (Figure 12B). To further explore the SAR of these inhibitors, they prepared and tested several analogs of hit compounds and identified three analog compounds with enhanced IC_50_. The first analog incorporates the benzothiazole, N-*tert* butyl pyrrole groups and the homopiperazine**,** with IC_50_ 1.2 µM, improved by 2 to 3-fold. In another analog, they replaced the t-butyl group by a hydrogen atom; resulting a substantially weakened inhibitor, with an IC_50_ of 61 µM. The *t*-butyl group with the heterocycle switched from the pyrrole to a thiophene, named the thiophene analog, *tBT-HBT*, provided the first nM non-covalent inhibitor of HhC cholesterolysis, with an IC_50_ of 300 nM (Figure 12C), ∼ 10-fold improvement in IC_50_ from the top hit screened library.

To understand the enzymatic mechanism of the inhibition, both *v*_*max*_ and *K*_*D*_ were measured and found to be affected by these compounds, indicating a mixed, non-competitive mechanism. This is further validated by photo-crosslinking of diazirine-cholesterol analog in the presence of inhibitor. These data suggest that cholesterol and non-competitive inhibitors can bind to HhC at the same time. To understand the structural basis of these compounds, molecular modeling and mutagenesis were carried out. It was found that both Hint and SRR residues are involved in inhibitor binding. Strikingly, the structures of HAC8 and tBT-HBT are very similar (Figure 12A), yet they have dramatically different effects. Establishing the structural basis of their activity difference in the future would be extremely beneficial to future drug screening efforts.

## Conclusion

In summary, great progress has been made in recent years in the areas of structural mechanisms and drug discovery of Hh autoprocessing. Inhibitors of Hh autoprocessing hold great promise for developing into a novel class of anti-cancer drugs for Hh ligand-dependent cancers, either as a single drug or in combination with other agents.

There are many important issues to be addressed in future studies of Hh autoprocessing and drug discovery. A key missing aspect is how Hh precursor interacts with the lipid bilayer of cell membrane and gains access to cholesterol, a highly insoluble membrane molecule. Understanding the precise structure activity relationship of Hh in its natural membrane environment will be key to determining the mechanism of Hh autoprocessing inhibitors and for developing cellular inhibitors of Hh autoprocessing. High resolution experimental structural studies of HhC and SRR are also missing, due to challenges associated with the aggregation and misfolding of HhC and SRR. Atomic resolution structure would be extremely valuable for understanding the precise details of how cholesterol and inhibitors interact with HhC. Although many compounds are efficient in inhibiting Hh autoprocessing *in vitro*, non-covalent compounds that are active in cellular assays are yet to be developed.
